# Evaluation of Sterilized Bioactive-Glass-Coated Magnetic Nanoparticles: Physicochemical Integrity and Biological Compatibility After Gamma Irradiation

**DOI:** 10.3390/pharmaceutics17081048

**Published:** 2025-08-12

**Authors:** João Gabriel Acioli de Siqueira, Ângela Leão Andrade, Rodrigo Ribeiro de Andrade, Pedro Igor Macário Viana, Lucas Resende Dutra Sousa, Paula Melo de Abreu Vieira, Gabriel Maia Vieira, Tatiane Cristine Silva de Almeida, Maximiliano Delany Martins, Samantha Roberta Machado de Oliveira, Flaviano dos Santos Martins, Marcelo Barbosa de Andrade, Rosana Zacarias Domingues, Alfredo Miranda de Goes, Guilherme Mattos Jardim Costa, Thalita Marcolan Valverde

**Affiliations:** 1 Departamento de Morfologia, Instituto de Ciências Biológicas, Universidade Federal de Minas Gerais (UFMG), Belo Horizonte 31270-901, MG, Brazil; 2 Departamento de Química, Instituto de Ciências Exatas e Biológicas, Universidade Federal de Ouro Preto (UFOP), Ouro Preto 35400-000, MG, Brazil; 3 Centro de Microscopia, Universidade Federal de Minas Gerais (UFMG), Belo Horizonte 31270-901, MG, Brazil; 4 Laboratório de Fitotecnologia, Escola de Farmácia, Universidade Federal de Ouro Preto (UFOP), Ouro Preto 35400-000, MG, Brazil; 5 Laboratório de Morfopatologia, Instituto de Ciências Biológicas, Universidade Federal de Ouro Preto (UFOP), Ouro Preto 35400-000, MG, Brazil; 6 Centro de Desenvolvimento da Tecnologia Nuclear (CDTN), Belo Horizonte 30161-970, MG, Brazil; gabrielmaiamg@gmail.com (G.M.V.); tatyanealmeyda@gmail.com (T.C.S.d.A.); mdm@cdtn.br (M.D.M.); 7 Departamento de Microbiologia, Instituto de Ciências Biológicas, Universidade Federal de Minas Gerais (UFMG), Belo Horizonte 31270-901, MG, Brazilflavianomartins@gmail.com (F.d.S.M.); 8 Departamento de Física, Instituto de Ciências Exatas e Biológicas, Universidade Federal de Ouro Preto (UFOP), Ouro Preto 35400-000, MG, Brazil; marcelo.barbosa@ufop.edu.br; 9 Departamento de Química, Instituto de Ciências Exatas, Universidade Federal de Minas Gerais (UFMG), Belo Horizonte 31270-901, MG, Brazil; 10 Departamento de Fisiologia e Biofísica, Instituto de Ciências Biológicas, Universidade Federal de Minas Gerais (UFMG), Belo Horizonte 31270-901, MG, Brazil

**Keywords:** nanoparticles, gamma irradiation, sterilization, bioactive glass, magnetic nanoparticles, biological safety

## Abstract

**Background/Objectives**: Gamma irradiation is a promising terminal sterilization method for nanoparticle-based biomedical systems. However, its potential effects on the physicochemical properties and biological performance of multifunctional nanomaterials must be carefully evaluated. This study aimed to assess the structural integrity, sterility, and cytocompatibility of magnetic nanoparticles (MNPs) and bioactive-glass-coated magnetic nanoparticles (MNPBGs), both based on magnetite (Fe_3_O_4_), after gamma irradiation. **Methods:** MNPs and MNPBGs were synthesized and subjected to gamma irradiation at 25 kGy, with additional doses explored in preliminary evaluations. Physicochemical characterizations were performed using XRD, TEM, SAED, and Raman spectroscopy. FTIR analyses were conducted on bioactive glass (BG) controls without magnetite. Sterility was evaluated via microbiological assays. Cytocompatibility and nitric oxide (NO) production were assessed using RAW 264.7 macrophages and Saos-2 osteosarcoma cells. Prussian blue staining was used to evaluate cellular uptake. **Results:** Gamma irradiation preserved the crystal structure, morphology, and size distribution of the nanoparticles. FTIR revealed only minor changes in the silicate network of BG, such as reduced intensity and slight shifting of Si-O-Si and Si-O-NBO bands, indicating limited radiation-induced structural rearrangement without affecting the material’s stability or cytocompatibility. Microbiological assays confirmed complete inhibition of microbial growth. All irradiated samples exhibited high cytocompatibility, with MNPBGs demonstrating enhanced biological responses. Notably, MNPBGs induced a more pronounced NO production in macrophages. Cellular uptake of nanoparticles by Saos-2 cells remained unaffected after irradiation. **Conclusions:** Gamma irradiation at 25 kGy is an effective sterilization strategy that maintains the structural and functional integrity of MNPs and MNPBGs. These findings support their safe use in sterile biomedical applications, particularly for bone-related therapies involving immunomodulation and drug delivery, with potential relevance for cancer treatment strategies such as osteosarcoma.

## 1. Introduction

Magnetic nanoparticles (MNPs), such as magnetite (Fe_3_O_4_) and maghemite (γ-Fe_2_O_3_), have shown great potential in various therapeutic applications, including drug delivery [1], tumor imaging [2] and magnetic hyperthermia in breast [3], prostate [4], and bone cancer [5,6]. Their functionality can be further enhanced by combining them with biomaterials, such as polymer matrices, tetramethylammonium hydroxide [7] or bioactive glass (BG) [5,8]. Among these, BG, a ceramic material known for promoting bone repair and wound healing [9], is particularly promising, making the integration of MNPs and BG a focus of increasing interest in research [5].

Given the critical role of these materials in advanced therapies, maintaining sterility is of utmost importance to prevent immune activation and mitigate health risks associated with contamination [10]. The presence of microorganisms, such as bacteria and fungi, can elicit inflammatory responses, potentially leading to systemic effects, including fever, organ dysfunction, or, in severe cases, sepsis [11]. These immune responses are triggered by the interaction of microbial components with host cells, resulting in the release of proinflammatory cytokines and the initiation of inflammatory cascades [12].

In this context, irradiation stands out as a highly effective sterilization method, capable of eliminating a broad spectrum of microorganisms while preserving the structural and functional integrity of sensitive materials, such as nanoparticles used in drug delivery [13,14]. Its efficiency, combined with the advantage of not requiring chemical agents, makes it particularly suitable for advanced therapeutic applications, where maintaining sterility, along with the biological functionality of components, is essential for both safety and efficacy [15].

Conventional sterilization methods are essential for ensuring material sterility but often introduce challenges that affect the integrity and physicochemical properties of advanced drug delivery platforms [16]. Among these methods, sterile filtration is widely employed for heat-sensitive solutions or suspensions. It utilizes a 0.22 µm membrane filter to remove contaminants while preserving chemical properties. Despite its advantages, filtration can clog membranes, reduce particle yield, or alter particle concentrations [17]. While synthesis optimization can address some of these issues, implementing such changes is not always practical or scalable.

Autoclaving is another widely used technique that relies on pressurized steam at approximately 120 °C for 20 min, providing a robust approach to eliminate microorganisms [18]. However, this technique is frequently associated with particle aggregation and an increase in particle size [18]. Additionally, heat-sensitive components, such as specific coatings or bioactive materials, may degrade under high temperatures, compromising their structural integrity and functional performance [19].

Among the commonly employed sterilization techniques are autoclaving, ultraviolet (UV) radiation, and gamma irradiation. Autoclaving is effective and widely used but can damage thermolabile materials. UV radiation, while nonthermal and simple to implement, has limited penetration depth, restricting its efficacy to surface sterilization of thin or transparent samples. In contrast, gamma irradiation combines deep penetration, broad-spectrum antimicrobial action, and compatibility with temperature-sensitive nanostructures. It avoids particle aggregation and yield loss due to filtration, is scalable for industrial use, and is effective against a wide range of pathogens, including bacteria, viruses, and fungi [20,21].

In this study, we investigated gamma irradiation at doses of 0 (nonirradiated control), 10, 15, 20, and 25 kGy. Among these, 25 kGy was selected for subsequent analyses because it meets the ISO 11137 guidelines to achieve a sterility assurance level (SAL) of 10^−6^ and represents the most clinically relevant and regulatory-approved condition for validating sterilized nanomaterials. Under these conditions, magnetic nanoparticles coated with bioactive glass (MNPBG), as well magnetic nanoparticles (MNPs), were subjected to irradiation. The aim was to evaluate gamma irradiation as an alternative sterilization method by analyzing its effects on the physicochemical properties and in vitro assay performances of these materials.

## 2. Materials and Methods

### 2.1. Reagents

The chemicals used were of analytical grade and did not require further purification. Ferric chloride hexahydrate (FeCl_3_·6H_2_O), hydrochloric acid (HCl), sodium sulfide (Na_2_SO_3_), ammonium hydroxide (NH_4_OH), tetraethyl orthosilicate (Si(OC_2_H_5_)_4_, TEOS), triethyl phosphate ((C_2_H_5_)_3_PO_4_, TEP), and calcium nitrate tetrahydrate (Ca(NO_3_)_2_·4H_2_O) were purchased from Sigma-Aldrich (St. Louis, MO, USA).

The following reagents were related to the biological assays: Brain–Heart Infusion (BHI) medium was purchased from Oxoid (Basingstoke, UK). Sabouraud Dextrose Agar was purchased from Merck Millipore (Burlington, VT, USA). The YPG medium was prepared using individual components: 1% yeast extract, 2% peptone, and 2% glucose (dextrose), all obtained from standard microbiological suppliers. *Geobacillus stearothermophilus* spores were obtained from an autoclaving sterility kit from Clean-Test (São Paulo, SP, Brazil). RPMI medium, Phosphate Buffered Saline (PBS) and Fetal Bovine Serum (FBS) were provided by Nova Biotecnologia (Cotia, São Paulo, Brazil). Streptomycin and penicillin were obtained from Thermo Fisher Scientific (Waltham, MA, USA), McCoy’s 5A medium and Trypsin-EDTA were purchased from Gibco (Grand Island, NY, USA). Lipopolysaccharides (LPS) was purchased from Sigma-Aldrich (St. Louis, MO, USA), and recombinant mouse interferon-gamma (IFN-γ) protein was obtained from Bio-Techne (Minneapolis, MN, USA). Saos-2 cells used in this study were obtained from the Rio de Janeiro Cell Bank (BCRJ, Rio de Janeiro, Brazil).

### 2.2. Synthesis

#### 2.2.1. Magnetic Nanoparticles

The synthesis of MNPs was carried out as described in previous works using a reduction–precipitation method [22]. A 30 mL solution of FeCl_3_·6H_2_O at 2 mol/L (dissolved in a 0.5 mol/L solution of HCl) was mixed with 20 mL of a 1 mol/L solution of Na_2_SO_3_ and 50.8 mL of NH_4_OH, and the final volume was adjusted to 800 mL with distilled deionized water. After combining ferric chloride and sodium sulfide, the resulting solution was kept under vacuum for 1 min, after which ammonium hydroxide was added quickly and the mixture was stirred vigorously for 30 min, maintaining the vacuum for the first minute. The resulting nonfunctionalized suspension was centrifuged at 2000 rpm for 3 min, and the transparent supernatant was discarded. This process was repeated five times, with resuspension in distilled water, which was discarded each time. This sample was called MNP.

The samples in each centrifuge tube were then suspended in 1 mL of a 25% solution of TMAOH. Typically, approximately 1 g of magnetite was present in each tube, and the resulting suspension was agitated vigorously until homogenized. Finally, the suspension was diluted tenfold with distilled water in preparation for coating with bioactive glass shells.

#### 2.2.2. Bioactive Glass and Coating

The bioactive glass used for coating the magnetic particles was prepared by hydrolysis and polycondensation of TEOS, TEP, and calcium nitrate tetrahydrate in the required amounts to obtain nominal compositions (mol%) of 63% silicon dioxide (SiO_2_), 34% calcium oxide (CaO), and 3% phosphorus pentoxide (P_2_O_5_).

For the coating, 1 mL of the final MNP suspension was diluted in 80 mL of absolute ethanol at room temperature, followed by ultrasonic agitation for 5 min. Then, 1 mL of TEOS and 20 mL of distilled water were added, and the mixture was stirred for 24 h. The resulting suspension was centrifuged at 12,800 rpm for 30 min. The supernatant was removed, and the precipitate was resuspended in 80 mL of ethanol and subjected to ultrasound. Then, 0.5 mL of TEOS, 20 mL of distilled water, and the appropriate proportions of CaO and P_2_O_5_ (as mentioned previously) were added.

The mixture was stirred for another 24 h and then centrifuged at 12,800 rpm for 30 min. The resulting powder was washed once acetone and three times with distilled water. It was then heated at a rate of 5 °C/min until it reached 700 °C, where it was maintained for 3 h before cooling. The resulting sample was named MNPBG, vacuum-packed, and stored under refrigerated conditions. Although heating at 700 °C likely eliminates microbial contaminants, subsequent handling steps, eventually carried out outside vacuum or sterile environments, may pose a risk of recontamination. To address this, gamma irradiation was employed as a terminal sterilization method, ensuring microbiological safety, particularly for sensitive formulations intended for biomedical use. Control samples without magnetite were also prepared using the same procedure and named BG. To minimize the risk of recontamination following irradiation, all post-treatment handling was carried out using sterile instruments under aseptic conditions. When manipulation outside laminar flow hoods was necessary, samples were immediately sealed in sterile containers or transferred to sterile biosafety cabinets prior to further use. Gloves and surfaces were disinfected with 70% ethanol, and all personnel wore appropriate protective gear.

### 2.3. Physicochemical Characterizations:

A variety of physicochemical characterizations were performed on MNPBG samples, both with and without gamma irradiation. When applicable, some characterizations were also conducted on MNP samples.

#### Structural Characterization and Morphological Analysis

Structural and morphological analyses were carried out on MNPBG samples, both with and without gamma irradiation, to investigate potential effects of the sterilization process. When applicable, comparisons were also made with MNP samples to evaluate the contribution of the bioactive glass coating.

X-ray diffraction (XRD) of MNPBG and MNP samples was performed using a Rigaku Ultima IV diffractometer (Tokyo, Japan), with a step size of 0.02°, scanning from 25° to 70° at a rate of 0.5°/min. Cu-Kα radiation (λ = 1.54 Å) was used as the source, operating at 40 kV and 30 mA. Diffraction patterns were refined using the Rietveld method implemented in the FULLPROF SUITE 2019 program (available at https://www.ill.eu/sites/fullprof/ (accessed on 3 October 2024).

Fourier-transform infrared (FT-IR) spectra of MNPBG and MNP samples were acquired using a Bruker Vertex 70v spectrometer (Billerica, MA, USA) equipped with an ATR-Platinum accessory with a diamond crystal. Raman spectra of MNP samples were obtained using a LabRAM HR Evolution spectrometer (Horiba France SAS, Villeneuve d’Ascq, France), equipped with a 600 g/mm grating and a liquid-nitrogen-cooled CCD detector, with excitation at 532 nm.

Scanning electron microscopy (SEM) was carried out using a FEI Quanta 3D FEG microscope (Carl Zeiss Microscopy, Jena, Germany), equipped with a backscattered electron detector for morphological evaluation and an energy-dispersive X-ray spectroscopy (EDS) system for elemental composition analysis.

Transmission electron microscopy (TEM), high-resolution TEM (HRTEM), and selected area electron diffraction (SAED) analyses were performed using a FEI Tecnai G2-20 SuperTwin microscope (Thermo Fisher Scientific-2006, Waltham, MA, USA) operating at 200 kV. The microscope was equipped with an Oxford silicon drift detector (SDD) for energy-dispersive X-ray spectroscopy (EDS). TEM and HRTEM images were used to assess particle morphology, size, and the presence of crystalline and amorphous phases. SAED patterns were used to confirm crystallographic structure and calculate lattice parameters. Size distribution was determined by measuring approximately 100 nanoparticles per sample from TEM images.

### 2.4. Biological Assays

#### 2.4.1. Microbial Assays

##### Microorganisms and Culture Conditions

*Candida albicans* (ATCC 10231—American Type Culture Collection, Arlington, VA, USA) were stored frozen at −80 °C in YPG Broth (1% yeast extract, 2% peptone, 2% glucose) with 20% glycerol. For the assay, it was cultivated in YPG medium at 37 °C for 24 h under constant agitation at 150 rpm (Esco OrbiCult™ IBS).

*Geobacillus stearothermophilus* spores (ATCC 7953—American Type Culture Collection, Arlington, VA, USA) were stored frozen at −80 °C in BHI medium with 20% glycerol as a cryoprotectant. For the assay, it was cultivated in BHI at 37 °C for 24 h without agitation.

##### Sample Preparation and Irradiation

Triplicates of 50 μL from a 150 μg∙mL^−1^ suspension of MNPBG, MNPs, and BG samples were prepared for each radiation dose and each tested microorganism species. Additionally, two positive control groups containing nonirradiated MNPBG, MNP, and BG samples were inoculated with either *C. albicans* or *G. stearothermophilus*. A negative control group consisting of nonirradiated and noninoculated MNPBG and MNP samples was also included.

Inoculation was carried out by mixing the samples with 25 μL of YPG or BHI medium containing *C. albicans* or *G. stearothermophilus*, respectively, in test tubes, resulting in a final sample concentration of 100 μg∙mL^−1^. The samples were then subjected to irradiation at doses of 10, 15, 20, and 25 kGy using a Cobalt-60 irradiation source (MDS Nordion, Ottawa, ON, Canada).

##### Microbial Growth and Colony Counting

After the incubation period, the samples inoculated with *C. albicans* were cultured on Petri dishes containing Sabouraud dextrose agar and incubated at 37 °C for 48 h. Samples inoculated with *G. stearothermophilus* were cultured on Petri dishes containing BHI agar under the same conditions. The negative control group was cultured on both Sabouraud and BHI media. Images of the Petri dishes were captured using a digital camera for qualitative analysis and colony counting.

#### 2.4.2. In Vitro Assays Using Eukaryotic Cell Lines

##### RAW 264.7 Cells

The murine macrophage cell line RAW 264.7 was cultured in RPMI 1640 medium supplemented with 10% fetal bovine serum (FBS), 100 μg·mL^−1^ streptomycin, and 500 U/mL penicillin. Cells were maintained at 37 °C in a humidified atmosphere containing 5% CO_2_. Subculturing was carried out at a 1:3 split ratio, with the culture medium replaced every 2 to 3 days.

##### Saos-2 Cells

The human osteosarcoma cell line Saos-2 was maintained in McCoy’s 5A medium supplemented with 10% FBS, 100 μg·mL^−1^ streptomycin, and 500 U/mL penicillin. Cells were incubated at 37 °C in a humidified atmosphere with 5% CO_2_. Subculturing was performed at a 1:4 split ratio, with medium replacement every 2 days.

#### 2.4.3. Sample Preparation

**Figure 1:**
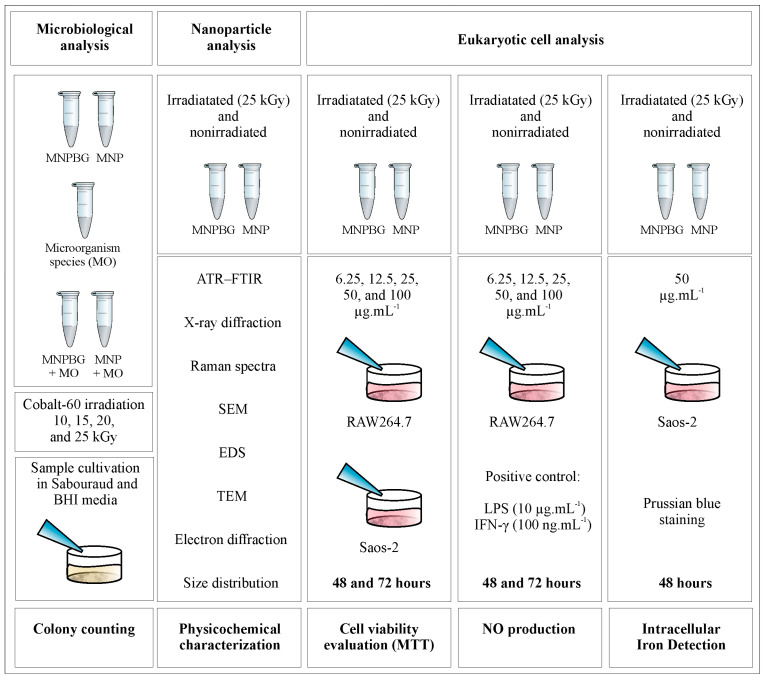
Experimental design for microbiological and eukaryotic cell analyses. Samples of MNP and MNPBG, irradiated or not, were tested for microbial growth (colony counting), physicochemically characterized, and evaluated in RAW264.7 and Saos-2 cells for cell viability (MTT assay), NO production, and intracellular iron detection (Prussian blue staining).

For biological assays involving eukaryotic cell cultures, the samples irradiated with 25 kGy were selected (Figure 1). These samples were dispersed in sterile water for injection and sonicated for 60 s to obtain the primary suspension. Final suspensions were then prepared at predefined concentrations of 6.25, 12.5, 25, 50, and 100 µg·mL^−1^, suitable for in vitro studies.

#### 2.4.4. Cell Viability Assay

The biocompatibility of MNPBG and MNP was evaluated using a (4,5–dimethylthiazol-2-yl)-2,5–diphenyl-2H-tetrazolium bromide (MTT) assay (Invitrogen^®^, Waltham, MA, USA), as described in [23]. After cell growth and expansion, a total of 1.5 × 10^4^ cells Saos-2 per well or 5 × 10^4^ Raw 264.7 cells per well were seeded into 48-well plates and cultured for 24 h. Subsequently, Saos-2 and Raw 264.7 cells were treated with either irradiated or nonirradiated MNPBG and MNP suspensions in 1× PBS at concentrations of 6.25, 12.5, 25, 50, and 100 µg·mL^−1^. The highest concentration (100 µg·mL^−1^) was included as a stress condition to evaluate the upper safety threshold of the nanoparticles. The viability control group received only 1× PBS, whereas the cytotoxicity control group was exposed to 0.05% (*v*/*v*) Triton™ X-100 for 5 min.

After 48 and 72 h of treatment, cells were incubated with 130 µL of a 5 mg·mL^−1^ MTT solution and 100 µL of growth medium. The reaction was stopped by adding 130 µL of a 10% of sodium lauryl sulfate (SDS) solution in 0.01 mol·L^−1^ HCl. All experiments were performed in triplicate. The mean absorbance of each sample group was normalized to the mean value of the viability control group. The percentage of cell viability was calculated using GraphPad Prism 6.0 software.

#### 2.4.5. Nitric Oxide Production Assay

RAW 264.7 cells were seeded at a density of 5 × 10^4^ cells per well and treated with either irradiated or nonirradiated MNPBG and MNP suspensions at concentrations of 6.25, 12.5, 25, 50, and 100 µg·mL^−1^. Cells were subsequently stimulated with LPS (10 μg.mL^−1^) or IFN-γ (100 ng·mL^−1^). Nitric oxide (NO) production, was assessed by measuring nitrite (NO_2_^−^) levels using the Griess reaction [24]. NO_2_^−^ concentrations were calculated based on a standard curve generated with sodium nitrite, and absorbance was measured at 570 nm using a spectrophotometer. All experiments were performed in triplicate.

#### 2.4.6. Prussian Blue Staining for Intracellular Iron Detection

The Prussian blue staining method was used to evaluate intracellular iron levels [25,26]. Saos-2 cells were cultured in McCoy’s 5A medium and seeded onto sterile coverslips placed in 6-well plates at a density of 3 × 10^5^ cells per well. Cells were treated with irradiated (25 kGy) and nonirradiated samples of MNP and MNPBG at a concentration of 50 μg.mL^−1^ for 48 h. After incubation, the cells were fixed with 4% paraformaldehyde for 30 min and washed twice with 1× PBS. A Prussian blue solution, prepared by mixing equal volumes of 13% hydrochloric acid and 10% potassium ferrocyanide, was added to each well and incubated for 30 min. The presence of ferric iron (Fe^3+^) was visualized as a blue pigment. Cells were then counterstained with neutral red for 5 min to enhance contrast between cellular structures and iron deposits. Microscopic analysis was performed using an optical microscope (Olympus BX60, Tokyo, Japan) at 1000× magnification.

#### 2.4.7. Statistical Analysis

Data from in vitro assays are expressed as mean ± standard deviation (SD) from at least three independent experiments. Statistical analyses were performed using GraphPad Prism 6.0 (GraphPad Software, USA). For nitric oxide (NO) production assays, one-way analysis of variance (ANOVA) was used, followed by Tukey’s post hoc test. For cell viability assays, two-way ANOVA was applied, also followed by Tukey’s post hoc test for multiple comparisons. Differences were considered statistically significant at *p* < 0.05. Because of the high number of pairwise comparisons, statistical significance is reported using a threshold approach (*p* < 0.05) rather than individual *p*-values. All data obtained were included in the analyses, and no outliers were excluded.

## 3. Results and Discussion

### 3.1. Microbiological Evaluation

A microbiological assay was performed to determine the minimum radiation dose required to prevent microbial growth on agar plates. As shown in Table 1, the highest dose tested, 25 kGy, was the only one at which no microbial growth was observed. Consequently, all samples used in subsequent physicochemical characterizations and in vitro assays were sterilized using 25 kGy of radiation. This finding corroborates well with international standards, as the ISO 11137-2 standard specifies methods to substantiate the use of 25 kGy as a sterilization dose to achieve a sterility assurance level (SAL) of 10^−6^ [27].

### 3.2. Physicochemical Characterizations

#### 3.2.1. Fourier-Transform Infrared Spectroscopy

To specifically evaluate the structural integrity of the silicate network, FTIR analysis was conducted only on bioactive glass (BG) control samples without magnetite. This approach was chosen to avoid spectral overlap with the strong Fe-O vibrational bands present in the MNP and MNPBG samples, which can obscure key features of the glass phase. FTIR spectroscopy is a sensitive technique for probing the local structure of silicate glasses, enabling the identification of changes in Si-O-Si vibrational modes, such as bond breakage and the formation of nonbridging oxygen groups (Si-O-NBO). These structural features play a key role in the biological response at the interface of bioactive materials when exposed to body fluids. This is mainly due to the presence of SiO_2_ as the major component, as the IR spectra are dominated by Si-O-Si stretching and bending modes, along with contributions from Si-O-NBO vibrations.

**Figure 2:**
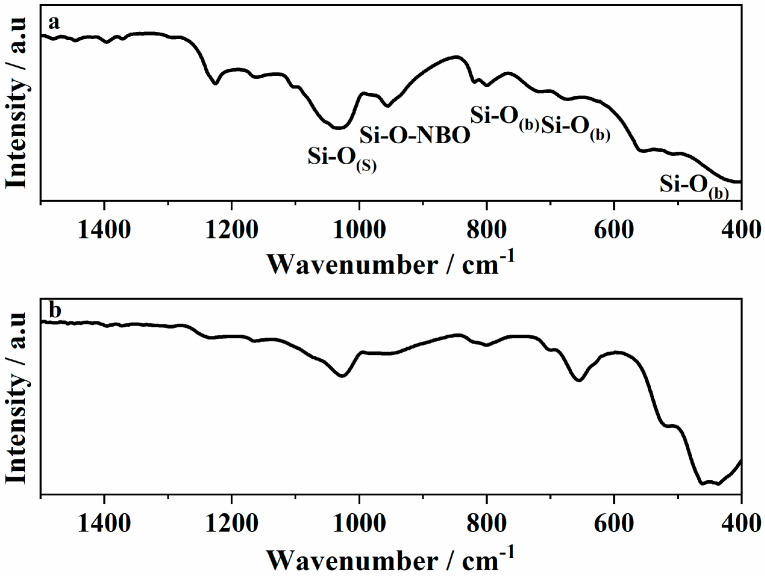
ATR–FTIR spectra of (**a**) BG 0 kGy and (**b**) BG 25 kGy.

The IR spectra illustrated in Figure 2a correspond to the prepared glass before gamma irradiation (BG 0 kGy sample). The IR bands were identified as follows: the Si-O_(s)_ stretching mode is in the range 1000–1300 cm^−1^. The Si-O_(b)_ bending mode is found around 800 cm^−1^, in the range 670–740 cm^−1^ and around 450–510 cm^−1^. The band between 890 and 975 cm^−1^ is associated with the Si-O_(s)_ with one nonbridging oxygen (Si-O-NBO) per SiO_4_ tetrahedron (Q^3^ groups) [28,29,30,31]. The small broad band at 1433 cm^−1^ can be attributed to the characteristic vibration modes of nonbridging P-O in PO_4_^3−^ groups.

After sterilization with gamma irradiation, significant differences were found in the silicon-oxygen bond vibrations regarding that of vitreous silica. The intensities of the bands associated to the Si-O_(s)_ and NBOs were decreased in the glass (Figure 2b). The bands related to the Si-O_(b)_ bonds, on the other hand, were broadened and shifted to lower wavenumbers. The increase in the intensity can be reflected in the change taking place in the glass structure after interaction with gamma irradiation and shifting of the bands towards a lower wavenumber.

It is assumed that gamma-radiation-induced structural disorders such as displacement, electron rearrangement, and radiolysis resulted in changes in the model parameters of building units such as bond position and/or bond angle [30,32]. A change in band intensity was observed, suggesting that the silica-based structures possessed a large surface area. Radiation-induced damage in glasses depends on several factors, including the radiation dose, the type and structure of the glass, and the presence of inherent defects. The damage caused by gamma irradiation in glass materials generally occurs through three primary mechanisms [33]: (1) densification, (2) structural deformation, and (3) electronic rearrangement. Our findings are consistent with those reported by other authors who also observed a reduction in the intensity of IR bands related to nonbridging oxygen species following gamma irradiation. For instance, Rai et al. [34] attributed the decrease in intensity to bond breaking or, more significantly, to a reorganization of the silicate network, with bond reformation playing a dominant role. This interpretation underscores the importance of network rearrangement processes in radiation-induced structural evolution. Similarly, Fayad et al. [35] associated the decrease in band intensity or broadening of bands with radiation-induced compaction of the glass matrix, which alters the vibrational environment of the structural units. These studies support the hypothesis that, depending on composition and structural features, gamma irradiation can lead to densification and reconfiguration of the glass network, ultimately affecting the detectability and definition of specific vibrational modes.

Although the observed changes in the FTIR spectra were subtle, such as decreased intensity and slight band shifts of Si-O-Si and Si-O-NBO vibrations, they may reflect minor local rearrangements within the silicate network. These modifications could influence surface reactivity, ion exchange kinetics, and the initial formation of hydroxyapatite in biological environments. However, the overall structural integrity of the glass was preserved, suggesting that the gamma irradiation process did not compromise the stability or potential bioactivity of the MNPBGs.

#### 3.2.2. X-Ray Diffraction

**Figure 3:**
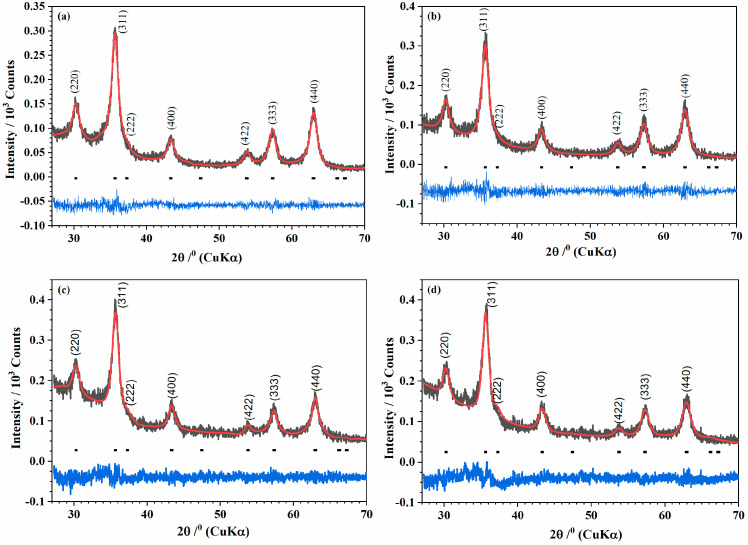
Powder X-ray diffraction patterns (Black) of the samples (**a**) MNP 0 kGy, (**b**) MNP 25 kGy, (**c**) MNPBG 0 kGy, and (**d**) MNPBG 25 kGy. The solid red lines correspond to the profiles fitted through Rietveld refinement. The solid blue lines correspond to the residual, or the difference between the calculated and experimental diffraction pattern.

The nanocrystalline structure of the iron oxide samples, irradiated and nonirradiated, was identified by powder XRD analysis. The broadening of the peaks was due to the small size of the ultrafine nanocrystallite. The Rietveld refinement of the XRD patterns was performed with $Fd\bar{3}m$ space groups for all samples. The pseudo-Voigt profile was used, and the resulting Rietveld profiles are displayed in Figure 3. The refinement showed that the lattice parameters of the nonirradiated samples increased after they were irradiated (Table 2). In addition, all samples, nonirradiated (MNP 0 kGy and MNPBG 0 kGy, Figure 3a and Figure 3c, respectively) and irradiated (MNP 25 kGy and MNPBG 25 kGy, Figure 3b and Figure 3d, respectively), corresponded to partially oxidized magnetite.

Stoichiometric magnetite, Fe_3_O_4_ (card no. 19-0629, space group $Fd\bar{3}m$), has an inverse spinel structure, with O^2–^ forming a face-centered cubic lattice and iron cations occupying interstitial sites. Ideally, in the unit cell of stoichiometric magnetite (Fe^3+^_A_[Fe^2+^Fe^3+^]_B_O_4_), all tetrahedral sites are occupied by Fe^3+^(“A” sites), and octahedral sites are occupied by both Fe^3+^ and Fe^2+^ (“B” sites) [36]. The ferrous to ferric ratio Fe^2+^/Fe^3+^ = 0.5. Maghemite (γ-Fe_2_O_3_, card no. 39-1346, space group P4_1_32) can be regarded as fully oxidized magnetite. In maghemite, oxygen vacancies form in the crystal structure to account for charge balance. Therefore, the partially oxidized magnetite Fe_3−x_O_4_ has stoichiometry between Fe_3_O_4_ and γ-Fe_2_O_3_. Accordingly, x can range from zero in stoichiometric magnetite to 1/3 in completely oxidized magnetite. The oxidation of Fe^II^ to Fe^III^ is accompanied by a decrease in the lattice cell parameter of stoichiometric magnetite from a = 8.396 Å to a = 8.349 Å in maghemite [37,38]. In our work, the calculated lattice cell parameters of samples are shown in Table 2. This behavior is explained by crystal distortion as a result of some ions that migrated into interstitial positions [39,40]. The increase in the lattice parameter of the irradiated samples is explained by the transformation [41] of the ferric ions, with smaller ionic radius (0.64 Å), to ferrous ions with larger ionic radius (0.78 Å). The creation of ferrous ions after γ-irradiation has been previously reported in Co–Zn ferrite 42 [42].

Iron (III) chloride in alkaline aqueous solution, when irradiated with gamma radiation, can form magnetite. Studies have shown that with increased irradiation, the obtained magnetite becomes more stoichiometric [43]. The explanation is that in an aqueous solution, gamma radiation produces a large number of solvated electrons. These can reduce the metal ions to lower oxidation states or even to neutral metal atoms [44]. Abedini also observed that increasing the radiation dose increased the Fe^2+^/Fe^3+^ ratio [45]. This was also observed in our work, concerning both samples, MNP and MNPBG. Although we worked with powders of the samples, the cubic lattice parameters increased after the irradiation of the samples. This result indicates that irradiation makes the Fe_3_O_4_ phase the preferred phase. The amorphous region that appeared between 20° and 35° (MNPBG 0 kGy and MNPBG 25 kGy samples depicted in Figure 3c and Figure 3d, respectively) is related to the amorphous silica network [46].

#### 3.2.3. Raman Spectroscopy

**Figure 4:**
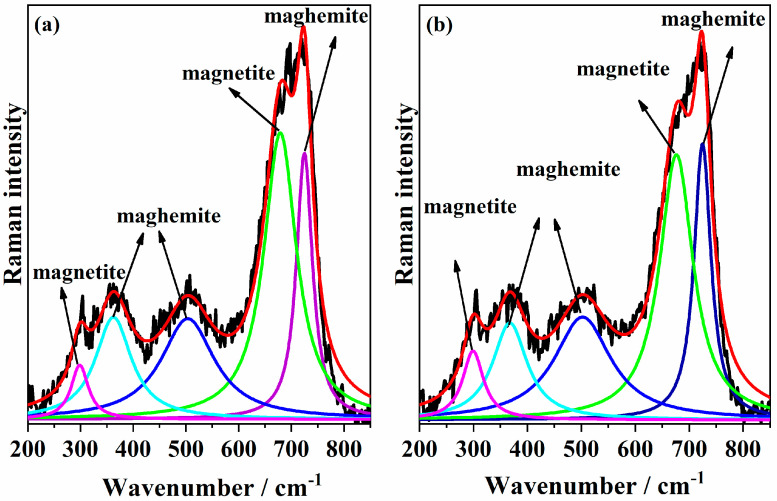
Deconvolution of Raman spectra of the samples (**a**) MNP 0 kGy and (**b**) MNP 25 kGy. The peaks highlighted in green and pink are attributed to magnetite, whereas the dark and light blue peaks correspond to maghemite.

The low-frequency region (150–800 cm^−1^) contains the characteristic Raman bands of iron oxides and is particularly interesting for the analysis of magnetite–maghemite content [47]. The Raman spectra of the samples MNP 0 kGy and MNP 25 kGy (Figure 4a and Figure 4b, respectively) were similar and confirmed the presence of magnetite and maghemite in the samples. The most intense band was decomposed into two Lorentzian components at 671 and 721 cm^−1^. The main A_1g_ mode of nanophase Fe_3_O_4_ was observed at 671 cm^−1^. The component at 721 cm^−1^ was assigned to the main band of nanophase γ-Fe_2_O_3_. The deconvolution of these two peaks showed that, after the sample irradiation, the peak related to magnetite was slightly more intense than that related to maghemite. This result confirms what was observed in the XRD.

According to the literature, the A_1_g vibrational mode of magnetite typically appears around 668 cm^−1^, although this value may vary depending on factors such as synthesis method and particle size [48]. Peak deconvolution was performed using Lorentzian peak shapes, yielding χ^2^ values of 0.9763 and 0.9774 for the MNP 0 kGy and MNP 25 kGy samples, respectively. Alternate fitting attempts using either a single or multiple bands were less effective in reproducing the experimental peak shape. In addition, the weak band observed at 298 cm^−1^ was attributed to Fe_3_O_4_, while the bands at 500 and 362 cm^−1^ are indicative of the presence of γ-Fe_2_O_3_.

#### 3.2.4. Scanning Electron Microscopy

**Figure 5:**
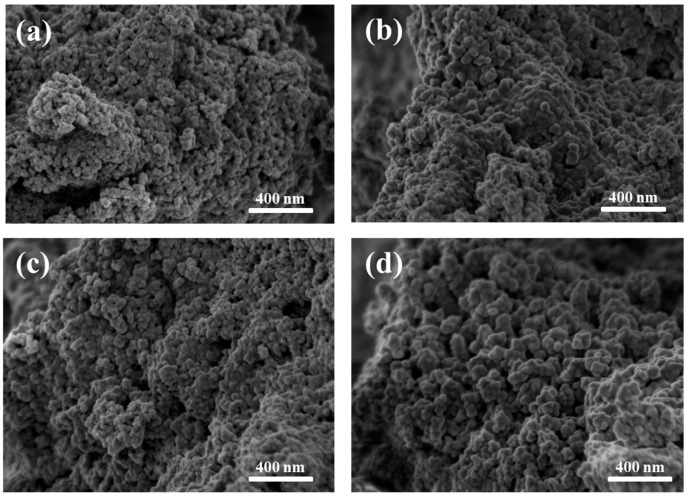
Scanning electron microscopy (SEM) micrographs acquired at 1 kV of (**a**) MNP 0 kGy, (**b**) MNP 25 kGy, (**c**) MNPBG 0 kGy, and (**d**) MNPBG 25 kGy. Nanoparticles appear as agglomerated clusters, with no major morphological differences among the samples.

The micrographs shown in Figure 5 reveal nanosized, spherical particles agglomerated in clusters. No significant morphological differences were observed between samples with and without bioactive glass coating, or between irradiated and non-irradiated groups (Figure 5a–d). Particle sizes were found to range from a few nanometers up to approximately 200 nm, which is consistent with the size range (10–200 nm) considered suitable for targeted drug/gene delivery systems and other biological applications [49].

**Figure 6:**
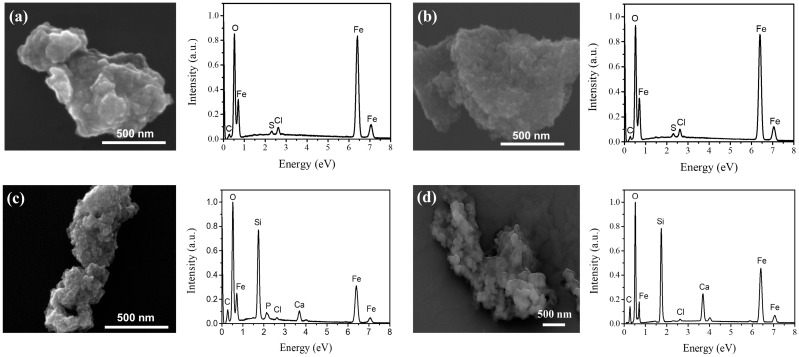
SEM images and corresponding EDS spectra of (**a**) MNP 0 kGy, (**b**) MNP 25 kGy, (**c**) MNPBG 0 kGy, and (**d**) MNPBG 25 kGy.

The morphological and elemental characteristics of MNP 0 kGy and MNP 25 kGy are presented in Figure 6a and Figure 6b, respectively. The left panels show micrographs of the samples, while the right panels display their corresponding EDS spectra, which exhibit intense peaks at energies corresponding to the emission lines of O Kα (0.525 KeV), Fe Lα (0.705 KeV) and Fe Kα (6.404 KeV), in accordance with the main composition of magnetite. Spurious peaks were detected with much less intensity (Cl and S) that can be related to residues coming from sodium sulfite and hydrochloric acid used for the MNP synthesis.

Micrographs and corresponding EDS spectra of MNPBG 0 kGy and MNPBG 25 kGy are shown in Figure 6c and Figure 6d, respectively. The left panels display the morphology of the bioactive-glass-coated samples, while the right panels present their EDS spectra. As observed, intense peaks for O and Fe are present. However, in contrast to uncoated MNPs, additional prominent peaks corresponding to the emission lines of Si Kα (1.740 KeV) and Ca Kα (3.691 KeV) confirmed the presence of the BG layer [50].

#### 3.2.5. Transmission Electron Microscopy

**Figure 7:**
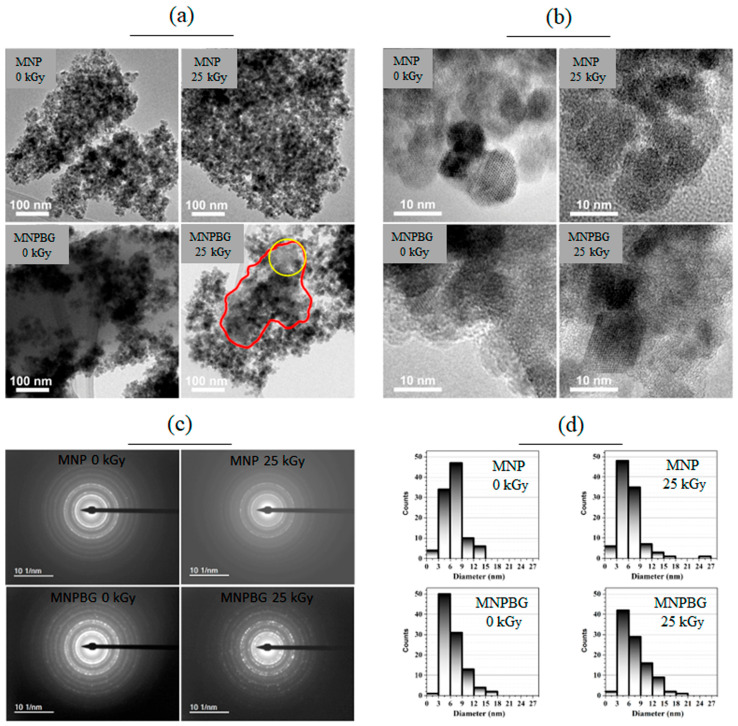
TEM analysis of MNP 0 kGy, MNP 25 kGy, MNPBG 0 kGy, and MNPBG 25 kGy samples. Bright-field images, HRTEM, SAED, and the histograms of the samples are shown in panels (**a**), (**b**), (**c**), and (**d**), respectively. In the MNPBG 25 kGy (panel **a**), the red circle highlights a homogeneous region corresponding to bioactive glass, while the yellow circle indicates a region containing both bioactive glass and nanoparticles.

Transmission electron microscopy (TEM) was employed to characterize the microstructure of the magnetic nanoparticles. The results are presented in Figure 7. Bright-field images shown in Figure 7a reveal the shape and size distribution of the analyzed nanoparticles. In these images, the nanoparticles are grouped in clusters, which is commonly observed in superparamagnetic systems because of magnetic dipole–dipole interactions and was also described by Andrade et al. for maghemite-based nanoparticles [22]. While the images of the MNP 0 kGy and MNP 25 kGy samples show only the nanoparticles, bioactive glass can be observed in the images of the MNPBG 0 kGy and MNPBG 25 kGy samples, with emphasis on MNPBG 0 kGy, in which the magnetic nanoparticles appear stacked on a plate-like structure aligned along the diagonal of the image. No relevant morphological differences were observed between irradiated and nonirradiated samples.

In the MNPBG 25 kGy image (Figure 7a), the bioactive glass is not as evident as in the MNPBG 0 kGy image, but it is still possible to observe it appearing as a homogeneous structure marked in the image (red circle). The stacking of nanoparticles on the bioactive glass resulted in dark spots, while the gray regions marked in the image by the yellow circle correspond to a region containing both bioactive glass and nanoparticles. This distribution pattern is consistent with previous reports describing the formation of compact aggregates during bioactive glass deposition attributed to magnetic interactions between particles that were not disrupted by the coating process [5].

To confirm the crystallinity of the nanoparticles and the presence of bioactive glass in the MNPBG 0 kGy and MNPBG 25 kGy samples, HRTEM images were acquired (Figure 7b). The images of the MNP 0 kGy and MNP 25 kGy samples showed clear lattice fringes, confirming the crystalline nature of the magnetic nanoparticles. In the MNPBG 0 kGy and MNPBG 25 kGy samples, crystalline regions attributed to the magnetic core were interspersed with amorphous regions, consistently with the presence of a disordered bioactive glass phase. This type of coexistence between crystalline ferrite and amorphous glass has been widely observed in bioactive nanocomposites, where the glass matrix provides structural support while maintaining magnetic functionality [51]. In the MNP 25 kGy sample, the amorphous region observed corresponds to the underlying carbon support film, as the nanoparticles were directly deposited on it, unlike in the other samples, where the particles themselves were thicker than the film. Notably, no loss of crystallinity or structural degradation was detected in any of the irradiated samples, indicating that gamma irradiation at 25 kGy did not compromise the crystal structure of the Fe_3_O_4_ phase. This finding aligns with previous studies reporting the structural resilience of magnetite-based systems subjected to similar irradiation doses [5].

Besides HRTEM, selected area electron diffraction (SAED) was also performed, allowing confirmation of the crystallography of the nanoparticles. This method enabled the identification of crystalline phases present in the analyzed clusters, contributing to a more comprehensive understanding of the structural features of the system, as well as the relationship between the morphology and crystallography of the magnetic nanoparticles and the bioactive glass matrix. Figure 7c presents the diffraction patterns obtained for all samples, along with the corresponding intensity profiles as a function of reciprocal distance. The SAED results indicate that the MNPs exhibited the same crystal structure across all samples and did not undergo structural changes upon irradiation. The lattice parameters were calculated from the diffraction ring distances observed in the SAED images, yielding the following values: MNP 0 kGy: (8.52 ± 0.03) Å, MNP 25 kGy: (8.51 ± 0.03) Å, MNPBG 0 kGy: (8.54 ± 0.04) Å, and MNPBG 25 kGy: (8.51 ± 0.02) Å. These values did not exhibit statistically significant differences, considering the measurement error, and furthermore, they were higher than those reported in the reference database (card no. 19-0629) and those determined by XRD. This discrepancy may have arisen from several factors, including dynamic electron–matter interactions that result in multiple scattering events and can shift the apparent positions of diffraction rings. Additionally, since SAED probes a localized region with a limited number of nanoparticles, it may be more sensitive to residual stress or structural relaxations within individual particles, which can affect the measured lattice parameters. Such effects are well documented in iron oxide nanostructures and may explain the small overestimations observed here [52].

In the final step, the size distribution of the nanoparticles was assessed from the TEM images by analyzing approximately 100 nanoparticles per sample. Figure 7d presents these results, showing histograms with very similar size distributions across all samples analyzed. The mean size and respective standard deviation (mean ± std dev) were calculated for the nanoparticle sets, yielding the following values: MNP 0 kGy: (7 ± 2) nm, MNP 25 kGy: (6 ± 3) nm, MNPBG 0 kGy: (6 ± 3) nm, and MNPBG 25 kGy: (7 ± 3) nm. No significant differences were observed between the samples. These results are consistent with previous reports indicating that gamma irradiation at comparable doses does not significantly affect the size or morphology of iron oxide nanoparticles [45].

### 3.3. In Vitro Assays

#### 3.3.1. Assessment of Saos-2 and RAW 264.7 Responses: Cytocompatibility and Nitric Oxide Production

Cytotoxicity assays are particularly important when evaluating materials that have undergone gamma irradiation, as this process may induce chemical or structural changes that affect biological safety. Such assessments help determine whether irradiation alters the material in a way that increases toxicity or compromises its compatibility with biological systems [21]. More broadly, cytotoxicity assays help determine concentration thresholds that avoid irreversible cellular damage, while also contributing to the overall assessment of biocompatibility in biomedical materials [53].

In this study, we assessed the cytotoxic effects of MNPs and MNPBGs using two cell lines: RAW 264.7 murine macrophages and Saos-2 human osteosarcoma cells. Macrophages are key regulators of inflammation and tissue responses to foreign materials. Therefore, evaluating the cytotoxicity of nanoparticles in macrophage cultures is essential to ensure biocompatibility, particularly in biomedical applications involving immune system interaction [54].

The Saos-2 cell line, derived from human osteosarcoma, was chosen because of its dual relevance: it serves both as a model of malignant bone cells and as a proxy for normal osteoblasts, given its preserved osteoblastic phenotype [55]. Studying nanoparticle interactions with osteosarcoma cells is especially relevant for bioactive-glass-based systems, which are traditionally used in bone repair but have also shown potential for targeted therapy in bone-related cancers [5].

To assess the biological safety of irradiated and non-irradiated nanoparticles, we performed cell viability assays under various experimental conditions, including different doses, exposure times, and treatment groups. Cell viability results were interpreted according to ISO 10993-5, an internationally recognized standard for the biological evaluation of medical devices. This guideline considers a material cytotoxic when it reduces cell viability to below 70%, indicating a biologically significant level of cell death in vitro [56]. This threshold was adopted in the present study as the benchmark for evaluating the cytocompatibility of the nanoparticles.

**Figure 8:**
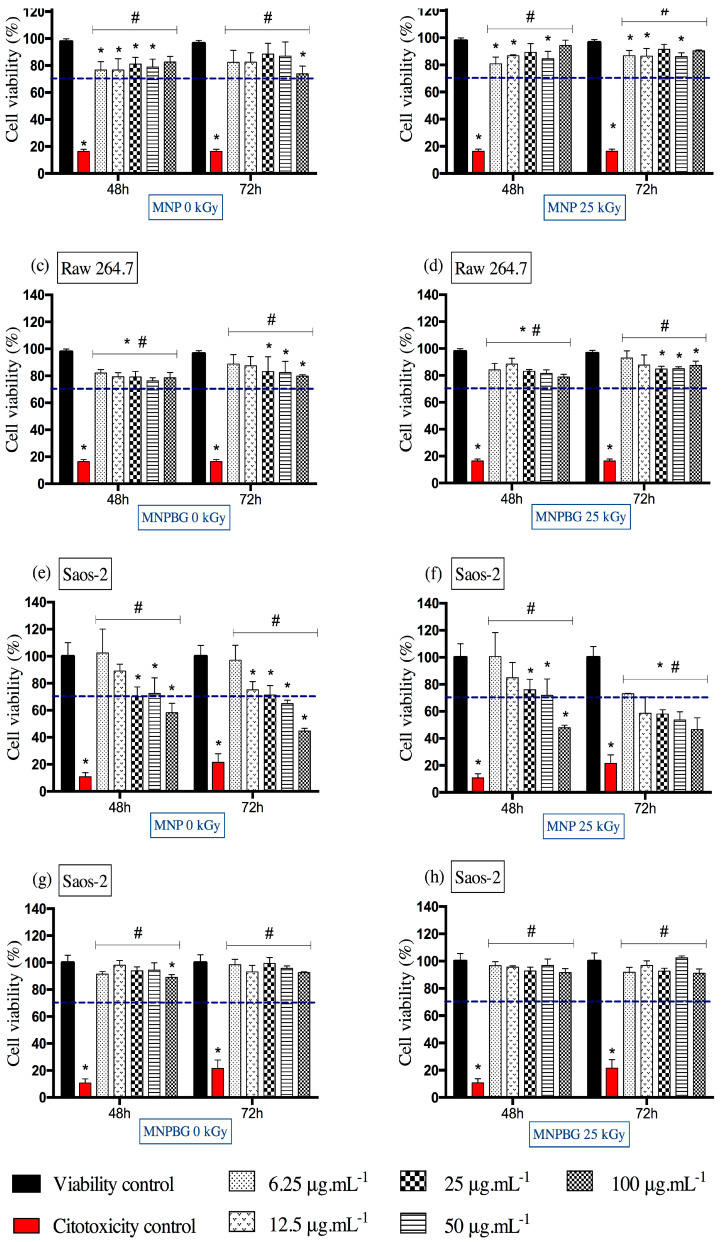
Cell viability assessed by the MTT assay in RAW 264.7 (**a**–**d**) and Saos-2 (**e**–**h**) cells following exposure to MNP and MNPBG nanoparticles, irradiated or not with 25 kGy, for 48 and 72 h at concentrations of 6.25, 12.5, 25, 50, and 100 µg.mL^−1^. Results are presented as mean ± standard deviation (SD) from triplicate experiments, with cell viability normalized to the control group treated with water for injection (viability control). Asterisks (*) indicate statistically significant differences compared with the viability control (*p* < 0.05). Hash symbols (♯) indicate significant differences compared with the cytotoxicity control (*p* < 0.05). Statistical analysis was performed using two-way ANOVA followed by Tukey’s post hoc test. The dashed line represents the 70% viability threshold acoording to ISO10993-5.

Based on this criterion, the results obtained for the RAW 264.7 macrophage cell line showed no evidence of cytotoxicity. As shown in Figure 8a–d, both MNPBGs and MNPs maintained cell viability above the 70% threshold under all tested conditions, regardless of exposure time and concentration. This observation is particularly relevant when compared with the findings of Kraus et al., who reported a dose- and time-dependent reduction in RAW 264.7 macrophage viability upon treatment with multicore iron oxide nanoparticles [54]. Their results also indicated increased oxidative stress, as evidenced by SOD activation and elevated ROS levels at higher concentrations. In contrast, our nanoparticles maintained high cell viability even after gamma irradiation, suggesting a more favorable cytocompatibility profile and minimal induction of oxidative stress pathways.

As shown in Figure 8e,f, the MNPs induced a more pronounced reduction in Saos-2 cell viability, particularly at higher concentrations and longer exposure times. In contrast, as shown in Figure 8g,h, the MNPBGs exhibited a more favorable cytocompatibility profile, maintaining cell viability above the 70% threshold under all tested conditions. Notably, gamma irradiation did not significantly impact the cytotoxicity of either nanoparticle type, indicating that the sterilization process preserved their biological safety. This result complements previous findings reporting good cytocompatibility of bioactive-glass-coated nanoparticles with Saos-2 cells at comparable concentrations and exposure durations (24 and 48 h), although those studies did not involve prior gamma irradiation [5].

The results from this study suggest that the bioactive glass coating may mitigate potential cytotoxic effects of the magnetic core, possibly by modulating the nanoparticle–cell interface. This is particularly relevant considering that, despite the use of a tumor-derived cell line, ensuring nanomaterials’ compatibility is essential to prevent off-target toxicity in surrounding healthy bone tissue [57]. The bioactive glass coating appears to act as a protective interface, reducing potential adverse effects from the magnetic core and enhancing overall cytocompatibility, even after gamma irradiation.

An additional in vitro assay performed was the measurement of nitric oxide (NO), a key inflammatory mediator produced by macrophages. NO plays a central role in proinflammatory responses, including cytotoxicity and modulation of cytokine-driven processes [58,59]. To assess its production, RAW 264.7 macrophages were exposed to proinflammatory cytokines, and NO levels were indirectly quantified. Unstimulated and LPS + IFN-γ-stimulated cells served as controls.

**Figure 9:**
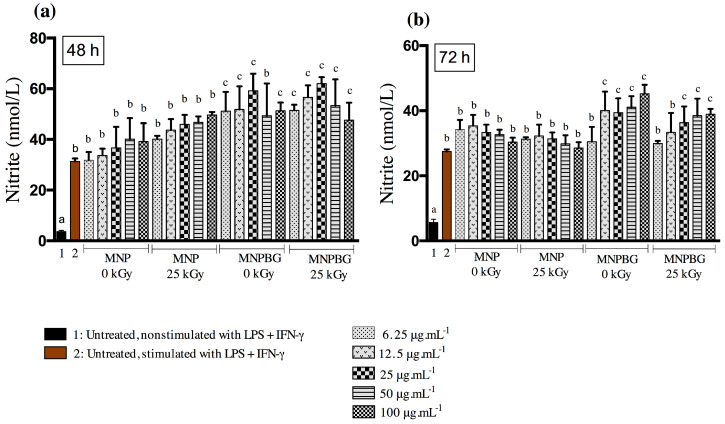
Nitric oxide (NO) production in RAW 264.7 cells after exposure to five different concentrations of MNP and MNPBG (6.25, 12.5, 25, 50, and 100 µg·mL^−1^) for (**a**) 48 h and (**b**) 72 h. Untreated and nonstimulated cells (without LPS + IFN-γ) served as the basal control (1), and untreated but stimulated cells (with LPS + IFN-γ) served as the stimulated control (2). Each bar represents the mean ± standard deviation (SD) from triplicate experiments. Bars labeled with the same letter are not significantly different (*p* > 0.05), while bars with different letters differ significantly, based on one-way ANOVA followed by Tukey’s post hoc test.

Despite the absence of cytotoxicity in Raw264.7 cells, treatment with MNPBG induced a higher NO response than uncoated MNPs at most concentrations, particularly at 6.25 to 25 μg·mL^−1^ and at 100 μg·mL^−1^, especially at the 48 h timepoint, regardless of gamma irradiation (Figure 9a,b). This suggests that the bioactive glass coating enhances immune activation. Given the plasticity of macrophages and their sensitivity to environmental stimuli, the increased NO production, often associated with M1 polarization via inducible nitric oxide synthase (iNOS) [60], is indicative of a functionally activated state. The higher nitric oxide (NO) production observed in RAW 264.7 macrophages exposed to MNPBGs, compared with uncoated MNPs, suggests that the bioactive glass coating actively contributes to the modulation of innate immune responses. This enhanced NO response may be attributed to the release of ionic products of calcium silicate from the bioactive silicate ceramics. These ionic products have been shown to modulate macrophage polarization, not only influencing inducible nitric oxide synthase (iNOS) pathways but promoting a shift toward the M2 phenotype. This shift involves downregulation of proinflammatory factors and M1 markers, along with upregulation of anti-inflammatory factors and M2 markers [61]. This M2 polarization is particularly relevant for bone regeneration, as M2 macrophages secrete anti-inflammatory cytokines and osteogenic growth factors, such as TGF-β, VEGF, and BMP-2, which enhance angiogenesis, recruit osteoprogenitor cells, and promote new bone formation [62]. Additionally, in the context of cancer therapy, NO has a dual role, potentially promoting tumor cell apoptosis at high concentrations while also modulating immune surveillance. These findings underscore the importance of further investigating the immunological impact of MNPBGs in vivo [63].

Furthermore, these results align with previous studies showing that iron oxide nanoparticles, such as ferumoxytol, can promote macrophage activation and support antitumor immunity through NO-related pathways [64]. The preservation of this response after gamma irradiation further reinforces the safety and translational potential of MNPBGs for biomedical applications, particularly those involving macrophage modulation.

#### 3.3.2. Prussian Blue Staining for Intracellular Iron Detection

**Figure 10:**
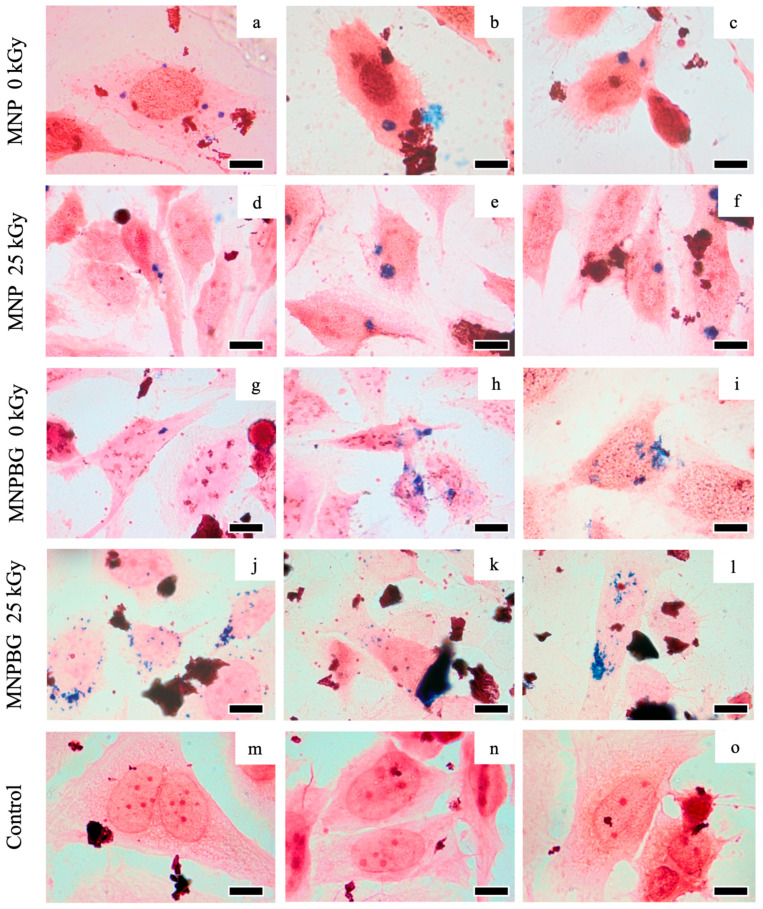
Prussian blue staining in Saos-2 cells for intracellular iron detection following exposure to different samples: MNP 0 kGy (**a**–**c**), MNP 25 kGy (**d**–**f**), MNPBG 0 kGy (**g**–**i**), and MNPBG 25 kGy (**j**–**l**), all at the target concentration of 50 µg.mL^−1^ for 48 h. The control group (**m**–**o**) was not exposed to nanoparticles and showed only neutral red counterstaining. Scale bar: 10 µm.

Nanoparticles were detected in all analyzed samples of MNP and MNPBG (Figure 10). The presence of blue staining in a human osteosarcoma cell line, the cell lineage Saos-2 (Figure 10a–l), indicates a positive result for intracellular iron detection. Ferricyanide, which reacts with Fe3+ from MNP 0 kGy (Figure 10a–c), MNP 25 kGy (Figure 10d–f), MNPBG 0 kGy (Figure 10g–i), and MNPBG 25 kGy (Figure 10j–l), resulted in a bluish stain in the cytoplasm of the cells 48 h after exposure. In contrast, only the counterstaining with neutral red was visible in the control group (Figure 10m–o), confirming the specificity of the Prussian blue reaction. Notably, it was possible to observe the uptake of irradiated versus nonirradiated nanoparticles, indicating that gamma sterilization at 25 kGy did not impair cellular internalization of the nanoparticle samples. This result reinforces the suitability of gamma irradiation as a sterilization method that preserves nanoparticle–cell interactions.

The Prussian blue technique proved to be a valuable and accessible method for detecting and visualizing the intracellular accumulation of iron-containing nanoparticles. Our findings align with previous studies reporting successful detection of iron oxide nanoparticles in vitro using this method in various cell types, such as macrophages [5] and fibroblasts [25]. The internalization of MNPBG in osteosarcoma cells is a promising result, as it suggests the potential for bioactive glass based nanoparticles to act as a therapeutic tool in bone cancer treatment, not only promoting bone regeneration but potentially carrying antitumor drugs to target cancer cells [65]. Therefore, expanding nanoparticle detection methodologies is essential, as it will pave the way for a future that associates antitumor drugs with MNPBG, potentially enhancing targeted treatment strategies.

## 4. Conclusions

This study demonstrated that gamma irradiation at 25 kGy is an effective terminal sterilization method for magnetic nanoparticles (MNPs) and bioactive-glass-coated magnetic nanoparticles (MNPBGs), preserving their physicochemical integrity and biological functionality. Structural analyses by XRD, Raman spectroscopy (for MNPs), TEM, and SAED confirmed that the crystal structure, morphology, and size distribution remained stable after irradiation. FTIR spectroscopy, performed on BG control samples without magnetite, revealed only minor rearrangements in the silicate network that did not compromise the integrity of the glass phase.

Microbiological assays validated the sterilization effectiveness, with complete inhibition of microbial growth. Biological assays using RAW 264.7 macrophages and Saos-2 osteosarcoma cells demonstrated high cytocompatibility, particularly in MNPBG samples. Furthermore, gamma-irradiated nanoparticles preserved their biological functionality, as evidenced by sustained nitric oxide production in macrophages, particularly in response to MNPBG, and efficient internalization by Saos-2 osteosarcoma cells. Altogether, these results confirm that gamma irradiation preserves both structural and functional properties of MNPBG nanoparticles, reinforcing their potential for sterile biomedical applications such as bone regeneration and targeted therapy. Although translational use requires further evaluation of long-term behavior, immune interactions, and regulatory compliance, these nanoparticles exhibit key properties that support their advancement toward clinical use. Their combined bone affinity and magnetic responsiveness make them strong candidates for future therapeutic strategies, including osteosarcoma treatment, where preliminary data already indicate selective cytotoxicity and encourage further investigation. Future studies may also explore their combination with standard chemotherapeutic agents (methotrexate, doxorubicin, and cisplatin) to assess potential synergistic effects in osteosarcoma treatment.

## Figures and Tables

**Table 1:** Agar plates were qualitatively inspected and recorded as positive (+) when any microbial colony growth was observed.

Sample (Dose)	*Candida albicans*	*Geobacillus stearothermophilus*
MNP (25 kGγ)	(−)	(−)
MNPBG (25 kGγ)	(−)	(−)
MNP (20 kGγ)	(−)	(−)
MNPBG (20 kGγ)	(−)	(+)
MNP (15 kGγ)	(−)	(−)
MNPBG (15 kGγ)	(−)	(+)
MNP (10 kGγ)	(+)	(+)
MNPBG (10 kGγ)	(−)	(+)
MNP (0 kGγ)	(+)	(+)
MNPBG (0 kGγ)	(+)	(+)

**Table 2:** Structural parameters and *Chi^2^* after Rietveld refinement for MNP 0 kGy, MNP 25 kGy, MNPBG 0 kGy, and MNPBG 25 kGy samples.

Parameter	Samples			
	MNP (0 kGy)	MNP (25 kGy)	MNPBG (0 kGy)	MNPBG (25 kGy)
a = b = c	8.351 ± 0.003	8.355 ± 0.003	8.350 ± 0.004	8.356 ± 0.003
*R_P_*	7.09	10.2	5.60	6.54
*R_WP_*	9.36	13.6	7.09	8.38
*S*	0.72	1.07	0.74	1.15
*R_B_*	1.73	4.18	4.07	6.61
*R_F_*	1.47	2.59	2.25	5.13
*Chi* ^2^	0.513	1.15	0.551	0.745

*R_P_* = profile R-factor; *R_WP_* = weighted profile R-factor; *S* = goodness-of-fit indicator (*S = R_WP_/Rexp*); *R_B_* = Bragg R-factor, based on integrated intensities; *R_F_* = structure factor R-factor, based on calculated and observed structure factors; *Chi^2^* = chi-square, statistical measure of the fit quality.

## Data Availability

The original contributions presented in this study are included in the article. Further inquiries can be directed to the corresponding authors.

## Abbreviations

The following abbreviations are used in this manuscript:

BG	Bioactive glass
BHI	Brain–heart infusion
EDS	Energy-dispersive X-ray spectroscopy
FBS	Fetal bovine serum
FTIR	Fourier-transform infrared
HCl	Hydrochloric acid
HRTEM	High-resolution TEM
IFN-γ	Interferon-gamma
iNOS	Inducible nitric oxide synthase
ISO	International Organization for Standardization
kGy	Kilogray
LPS	Lipopolysaccharides
MNP	Magnetic nanoparticles
MNPBG	Bioactive glass-coated magnetic nanoparticles
MO	Microorganism species
MTT	(4,5-dimethylthiazol-2-yl)-2,5-diphenyl-2H-tetrazolium bromide
PBS	Phosphate-buffered saline
RPMI	Roswell Park Memorial Institute (medium)
SAL	Sterility assurance level
SAED	Selected area electron diffraction
SD	Standard deviation
SDD	Silicon drift detector
SDS	Sodium lauryl sulfate
SEM	Scanning electron microscopy
Si	Silicon
TEM	Transmission electron microscopy
TEOS	Tetraethyl orthosilicate
TEP	Triethyl phosphate
TMAOH	Tetramethylammonium hydroxide
XRD	X-ray diffraction
YPG	Yeast extract, peptone, glucose (dextrose)
